# Does Branched-Chain Amino Acids (BCAAs) Supplementation Attenuate Muscle Damage Markers and Soreness after Resistance Exercise in Trained Males? A Meta-Analysis of Randomized Controlled Trials

**DOI:** 10.3390/nu13061880

**Published:** 2021-05-31

**Authors:** Chutimon Khemtong, Chia-Hua Kuo, Chih-Yen Chen, Salvador J. Jaime, Giancarlo Condello

**Affiliations:** 1 Institute of Sports Sciences, University of Taipei, 101 Zhongcheng Rd. Section 2, Shilin District, Taipei 111, Taiwan; tmedmet.md_sci3@hotmail.com (C.K.); kuochiahua@gmail.com (C.-H.K.); 2 Division of Gastroenterology and Hepatology, Department of Medicine, Taipei Veterans General Hospital, Taipei 112, Taiwan; chency@vghtpe.gov.tw; 3 Faculty of Medicine and Institute of Emergency and Critical Medicine, National Yang Ming Chiao Tung University College Medicine, Taipei 112, Taiwan; 4 Department of Exercise and Sport Science, University of Wisconsin-La Crosse, La Crosse, WI 54601, USA; sjaime@uwlax.edu; 5 Graduate Institute of Sports Training, University of Taipei, 101 Zhongcheng Rd. Section 2, Shilin District, Taipei 111, Taiwan

**Keywords:** branched-chain amino acids, muscle damage, muscle soreness, creatine kinase, lactate dehydrogenase, resistance exercise, inflammatory response, meta-analysis

## Abstract

Previous studies have reported the positive effects of branched-chain amino acids (BCAAs) supplementation on lowering plasma markers of muscle damage and subjective soreness after resistance exercise. However, a variety of factors can potentially moderate its efficacy. This meta-analysis aimed to summarize the evidence regarding the effect of BCAAs supplementation on plasma muscle damage markers and soreness after resistance exercise in only trained males, by considering the plasma lactate dehydrogenase (LDH) and creatine kinase (CK). Randomized controlled trials were identified through a computerized literature search for the period 2010–2020. The pooled data were analyzed with the random-effects model and heterogeneity using *I*^2^. Cochrane Collaboration tools was used for the assessment of risk of bias. Nine studies met the inclusion criteria. A positive effect was found for CK at <24, 24, and 48 h after exercise and for muscle soreness at <24 h only. However, the positive effect was not evident for plasma LDH at any follow-up time. Different outcomes for post-exercise responses may suggest that BCAAs supplementation can attenuate muscle damage and ameliorate muscle soreness after resistance exercise in trained males.

## 1. Introduction

Resistance exercise is commonly used by general population and athletes to increase muscular strength, endurance, power, and muscle mass. It can be executed across the three muscle contractions, eccentric, concentric, and isometric, in isolation or combination. Eccentric muscle contraction has been postulated to induce a higher magnitude of muscle damage compared with concentric and isometric ones [1,2], in particular when unaccustomed eccentric exercises with greater force production [3] and fast angular velocities [4] are performed. The proposed model of exercise-induced muscle damage (EIMD) includes two phases, the primary damage resulting from the mechanical stress during the exercise bout, and the subsequent secondary damage involving the loss of membrane integrity at the sarcoplasmic reticulum and causing the leakage of intramuscular proteins from the muscle sarcolemma into the blood for several days after exercise [5]. This secondary damage can be associated with the inflammatory responses, which are divided in early and late stages, encompassing satellite cells activation and proliferation and terminal differentiation and growth, respectively [6]. The possible consequences of EIMD are disruption of intracellular muscle structure, sarcolemma and extracellular matrix, prolonged impairment of muscle function, delayed-onset muscle soreness (DOMS), stiffness, and swelling lasting for several days [7].

The inflammation status can be identified by the indirect inflammatory biomarkers elevated into the blood circulation, such as lactate dehydrogenase (LDH) and creatine kinase (CK) [8,9], myoglobin, and cytokines [1,10]. Plasma LDH elevation occurs immediately after exercise before CK increases. Differently, plasma CK may peak anywhere from 24 to 96 h [11,12]. The different response patterns of these two markers may correspond to different stages of inflammation (early and late stage) before resolution of inflammation [6]. Although exercise-induced inflammation is essential for adaptive remodeling [13], subsequent exercise sessions could be compromised by residual muscle soreness, restriction of movement, and reduced capacity. Therefore, several strategies have been suggested to mitigate these negative consequences from EIMD, including massage, cryotherapy, stretching, non-steroidal anti-inflammatory drugs, and nutritional strategies [5].

Considering the nutritional strategies, dietary protein provides amino acids to stimulate muscle protein synthesis in order to support the inflammatory process and muscle regeneration after exercise [14,15]. Due to the existence of several protein sources, the composition of amino acids and bioavailability of each source may influence the understanding of protein quality and the capacity of the diet to support muscle protein synthesis [15,16]. In particular, dietary protein sources vary for the branched-chain amino acids (BCAAs) content, which ranges from 14% (potatoes) to 26% (milk) and is considered an important factor for stimulating muscle protein synthesis and promoting muscle growth [15]. Moreover, the three components of BCAAs (i.e., leucine, isoleucine, and valine) are primarily catabolized in the skeletal muscles, whereas other amino acids are catabolized in the liver [17]. Consequently, the protein turnover in the muscle cells can be directly regulated by BCAAs to counteract the catabolic and anti-anabolic effects produced by EIMD [18]. Specifically, leucine has been identified as a key regulator of mTOR signaling and translation initiation [15]. Therefore, among the possible protein sources, whey protein, BCAAs, and leucine-enriched essential amino acids are suggested for the supplementation strategy due to the high availability of amino acids (especially leucine) for the promotion of muscle protein synthesis [15,18,19]. However, BCAAs have the advantages to be lower in calories, omitting gluconeogenesis, and preventing augmented kidney workload compared with high protein diet, like dietary or whey protein [20]. Therefore, the use of BCAAs as supplementation strategy becomes popular among general population and athletes due to its ability in attenuating the negative symptoms of EIMD [21].

Recently, the effects of BCAAs on the EIMD mitigation and muscle soreness have been widely investigated under different exercise conditions and populations, even though heterogeneity exists among outcomes and methodological quality [22]. A summary of evidence is available from previous meta-analyses [23,24,25]. Evidence demonstrates the potential positive effect of BCAAs supplementation on muscle damage, muscle soreness and function [23], fatigue substances, energy metabolites and muscle soreness substances [24], and muscle damage [25]. The previous meta-analyses have been conducted with broad eligibility criteria in terms of gender (both male and female participants), training status (trained and untrained populations), type of exercise (endurance, resistance, or specific sports), and supplementation interventions (BCAAs combined with other amino acids and vitamins). Although this evidence can still be informative, the generalizability of the findings could be influenced by the effects of potential moderators, hence a refined selection of eligibility criteria could narrow the understanding of the effect of BCAAs supplementation.

Therefore, the purpose of this article was to conduct a meta-analysis of studies investigating the effects of BCAAs supplementation on the plasma muscle damage markers and soreness after resistance exercise in trained males. The evaluation of markers LDH and CK may help to provide insight into the effects of BCAAs supplementation related to the early and late stages of the inflammation. Furthermore, female hormones are fluctuating with time and estrogen has been shown to influence the exercise-induced response in plasma muscle damage markers [26]. To avoid the potential influence of sex hormone, the present meta-analysis only included studies on trained males.

## 2. Materials and Methods

### 2.1. Study Protocol

The systematic review with meta-analysis was conducted according to the Preferred Reporting Items for Systematic Reviews and Meta-Analysis (PRISMA) guidelines [27]. This review was registered with the International Prospective Register for Systematic Reviews (PROSPERO—registration number: CRD42021231999; 19 February 2021). The definition of the inclusion criteria followed the PICOS model [28] (Table 1).

### 2.2. Search Strategy and Study Selection

The searching strategy was conducted using the online databases and their related thesaurus SportDiscus, google scholar, PubMed, BASE, Scopus, and Semantic scholar for the period from January 2010 to December 2020. The literature search was conducted using the following keywords, as free text terms and thesaurus terms: (Branched-chain amino acid OR BCAA OR BCAAs) AND (exercise OR training) AND (muscle damage OR soreness OR recovery). Additionally, the reference lists of the included studies were reviewed to identify other eligible articles.

The literature search was performed independently by two reviewers (CK and GC) and inconsistencies were solved by consensus. Titles and abstracts generated by the literature search were firstly reviewed. Abstracts without enough information regarding the eligibility criteria were retrieved for full-text evaluation. Full-text articles for those potentially eligible included in the meta-analysis were obtained and were subsequently screened for relevance using the eligibility criteria.

### 2.3. Eligibility Criteria

Studies were included in this meta-analysis if they met the following inclusion criteria: (1) full-text, peer-reviewed articles published in English; (2) randomized controlled trial (parallel and crossover study design) on human participants exploring the effects of BCAAs supplementation on muscle damage and/or soreness after resistance exercise; (3) BCAAs supplementation “before-exercise” and “before and after-exercise”, including BCAAs and carbohydrate or sweetener (dextrose, glucose, and others); (4) the control group with either resistance exercise alone (without supplementation) or exercise combined with non-protein placebo supplementation; (5) trained males or athletes participants with age between 18–25 years; (6) resistance exercise intervention; (7) any measurement of muscle damage and soreness at several follow-up times (i.e., <24, 24, 48, 72, 96 h).

The following exclusion criteria were considered: (1) the intervention aimed to treat a specific disease or medical condition; (2) co-ingestion of BCAAs supplementation with other essential amino acids; (3) co-ingestion of BCAAs supplementation with other agents (e.g., creatine, β-HMB, or testosterone-enhancing compounds); no information regarding the participants’ mean age.

### 2.4. Data Extraction

Using a standardized assessment sheet, two investigators (CK and GC) independently extracted relevant data: study identifiers (i.e., author identification, country of study, year of publication), participants’ characteristics (i.e., number, age, gender, body mass, training status), BCAAs supplementation (i.e., dose, timing), placebo/control supplementation (i.e., type, dose, timing), resistance exercise intervention (exercise mode, volume, and intensity), muscle damage and soreness outcomes (i.e., CK, LDH, VAS) at several follow-up times (i.e., <24, 24, 48, 72, 96 h).

Where data were not available in table, Web-based plot digitizer 4.3 (https://automeris.io/WebPlotDigitizer (accessed on 10 January 2021) and Foxit reader (Foxit Software Inc. Fremont, CA, United States) were used to extract the data from graph [29].

### 2.5. Risk of Bias and Study Quality Assessment

The Cochrane Collaboration tools [30] was used to assess the risk of bias of randomized controlled trial studies. This tool evaluates the random sequence generation, allocation concealment, blinding of participants and personnel, blinding of outcomes assessment, incomplete outcome data, selective outcome reporting, and other domains others which are not covered in the above. Each study was labelled as either a low risk of bias, a high risk of bias, or an unclear risk of bias. The data included in the meta-analyses were restricted to studies with less than two reported high-risk domains.

### 2.6. Statistical Analysis

The random-effects meta-analysis was performed using Review Manager software (RevMan 5.3; Cochrane Collaboration, Oxford, UK). Mean and standard deviation (SD) of the pre- and any post-intervention follow-up were obtained from the original studies. The mean differences and 95% confidence intervals (95% CIs) were calculated for continuous data of included studies. In addition, the changes of standard deviation (ΔSD) of the data in each study was calculated using formula according to the Cochrane Handbook for Systematic Reviews of Interventions [31]:

(1) $$\Delta SD=\sqrt{\left(SDpre^{2}+\mathrm{SDpos}t^{2}-2\times \mathrm{corr}\times \mathrm{SDpre}\times \mathrm{SDpost}\right)}$$

A correlation coefficient of 0.8 was assumed. Forest plots were generated to show the mean, SD, sample size for experimental and control group and mean differences and 95% CIs. Heterogeneity among studies was evaluated through *I*^2^ statistics, the Cochrane Chi square (χ^2^), and the between-study variance using the tau-square (τ^2^). The heterogeneity thresholds were *I*^2^ = 25% (low magnitude), *I*^2^ = 50% (moderate magnitude), and *I*^2^ = 75% (high magnitude) [32]. A *p* value < 0.1 for χ^2^ was defined as indicating the presence of heterogeneity. A τ^2^ > 1 suggested the presence of substantial statistical heterogeneity. The level of statistical significance was set at *p* < 0.05.

## 3. Results

### 3.1. Literature Search

**Figure 1:**
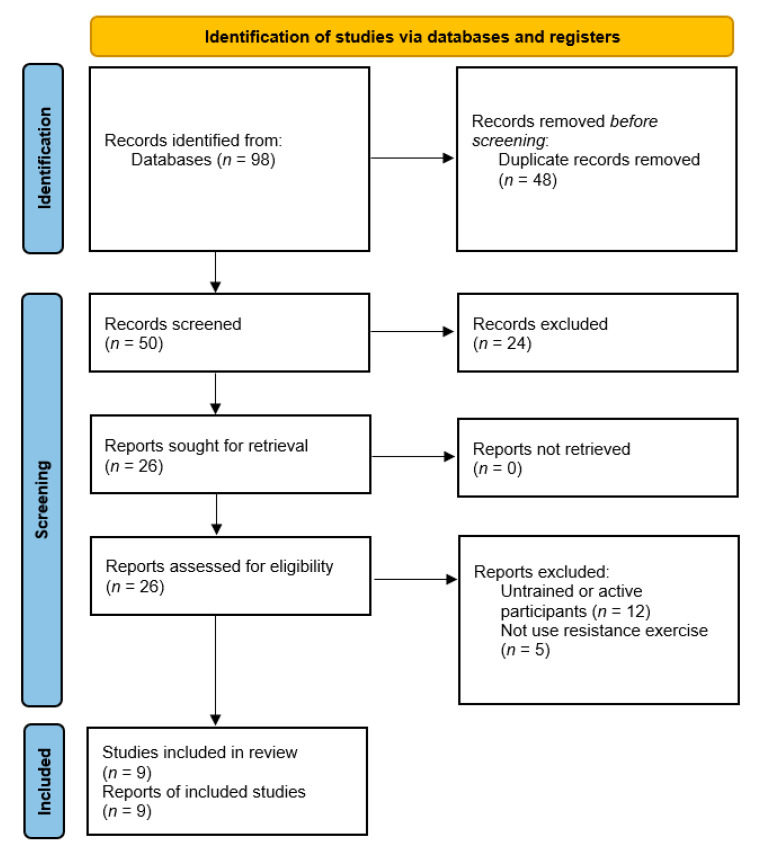
PRISMA flowchart of searching strategy and studies selection process.

The initial electronic search identified 79 eligible articles from the online databases together with 19 articles identified through the references lists of selected studies resulting in a total of 98 articles. After the removal of 48 duplicated articles, 50 articles were screened based on the title and abstract. A total 26 full-text articles were assessed in next stage, but 17 of them were then excluded due to not complying the eligibility criteria. As the result, a total of nine articles met the inclusion criteria and were included in the quantitative analysis (Figure 1).

The details of study characteristics are summarized in Table 2. The included studies ranged from 2011 to 2018 [33,34,35,36,37,38,39,40,41]. The study design was randomized controlled trial, of which parallel design for 7 studies [33,34,35,36,39,40,41] and crossover design for 2 studies [37,38]. Participants from the nine studies were either single [33,36,37,38] or double blinded [34,35,39,40,41]. 

### 3.2. Risk of Bias Assessment

**Figure 2:**
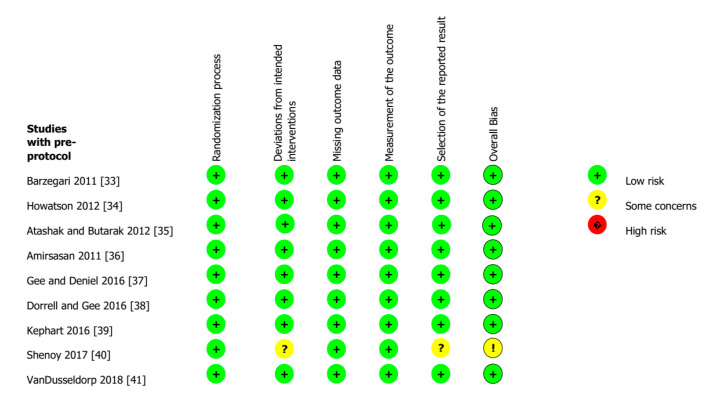
Risk of bias assessment. Green circle = low risk of bias; yellow circle = unclear risk of bias; red circle = high risk of bias.

Risk of bias assessment for each included study is presented in Figure 2. No evidence for publications bias was found.

### 3.3. Studies Characteristics

The total number of participants from all included studies was 278 with age ranging from 21.5 to 24.7 years and mean body mass ranging from 61.7 to 86.4 kg. Participants were declared to be resistance-trained males [37,38,39,41] and specific-sports athletes of soccer and rugby [34,35], wrestling [33,36], and road cycling [40].

### 3.4. BCAAs Supplementation

BCAAs supplementation was applied for a period ranging from 1 to 28 days, using the following strategies: at the pre-load only [40,41], at the exercise day only [35,37,38], at pre-load and exercise day [34], on the exercise day and recovery period [39], and at pre-load, exercise day and recovery period [33,36]. Most of the included studies were supplemented with a BCAAs ratio of 2:1:1 (leucine: isoleucine: valine) [33,34,35,36,37,38,40] whereas the BCAAs in the ratio 3:1:2 was used in two studies [39,41]. The dosage of BCAAs was between 0.20 to 1.76 g/kg of body weight and between 12 to 260 g.

### 3.5. Resistance Exercise Intervention

The resistance exercise interventions included multi-joint exercises with barbell [33,35,36,37,38], back squat [39], drop jump [34,40], and combination of squat and split jump [41]. The intensity of resistance exercise was performed between 70% 1RM to 100% 1RM. The volume of exercise was designed between three sets to volitional exhaustion and between 5 to 20 repetitions.

### 3.6. Muscle Damage and Soreness Outcomes

Muscle damage was measured with LDH in two studies [33,36] and CK in 6 studies [33,36,40,41], whilst five studies measured muscle soreness with VAS [34,37,38,39,41]. All included studies reported multiple follow-up times from <24 to 96 h after exercise. Three studies showed the data at <24 h after exercise with the combination of several follow-up times including post-exercise immediately [35,38], 1 and 2 h [35], and 4 h [41]. However, only the data from <24 until 48 h were further analyzed, since only two studies measured at 72 h [41] and 96 h [34]. 

### 3.7. Effect of BCAAs Supplementation on Muscle Damage and Soreness Outcomes

**Figure 3:**
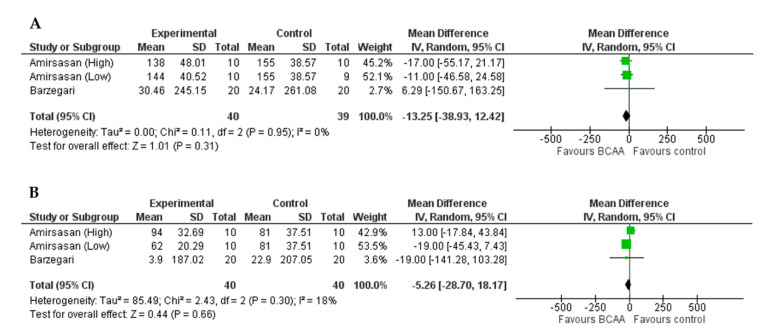
Forest plots of the effect of BCAAs on LDH compared to control group at 24 h (**A**) and 48 h (**B**).

No effect of BCAAs supplementation on LDH outcome emerged at both 24 h (MD = −13.25 U/L, 95%CI = −38.93, 12.42, *p* = 0.31, Figure 3A) and 48 h (MD = −5.26 U/L, 95%CI = −28.70, 18.17, *p* = 0.66, Figure 3B).

**Figure 4:**
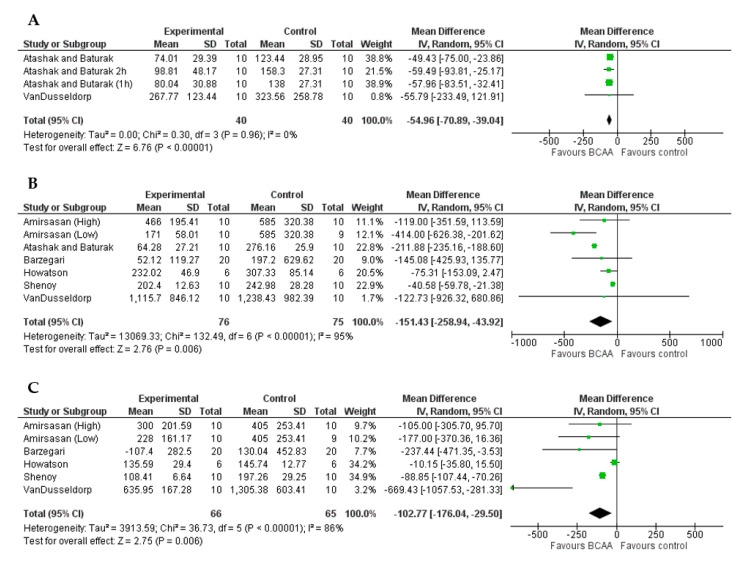
Forest plots of the effect of BCAAs on CK compared to control group at <24 h (**A**), 24 h (**B**), and 48 h (**C**).

A positive effect of BCAAs supplementation on CK outcome was found at <24 h (MD = −54.96 IU/L, 95%CI = −70.89, −39.04, *p* < 0.001, Figure 4A), 24 h (MD = −151.43 IU/L, 95%CI = −258.94, −43.92, *p* = 0.006, Figure 4B), and 48 h (MD = −102.77 IU/L, 95%CI = −176.04, −29.50, *p* = 0.006, Figure 4C). However, at both 24 and 48 h the variability between studies has been found to be high in magnitude (*I*^2^ > 75%).

**Figure 5:**
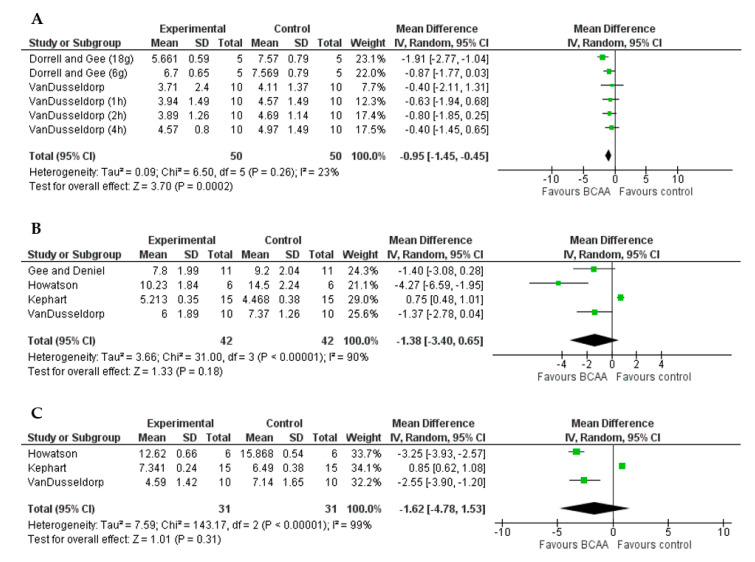
Forest plots of the effect of BCAAs on VAS compared to control group at <24 h (**A**), 24 h (**B**), and 48 h (**C**).

A positive effect of BCAAs supplementation on VAS was found at <24 h (MD = −0.95, 95%CI = −1.45, −0.45, *p* = 0.002, Figure 5A), but no at 24 h (MD = −1.38, 95%CI = −3.40, 0.65, *p* = 0.18, Figure 5B) and 48 h (MD = −1.62, 95%CI = −4.78, 1.53, *p* = 0.31, Figure 5C). Moreover, the variability at <24 h has been considered low in magnitude (*I*^2^ < 25%).

## 4. Discussion

This meta-analysis had the purpose to summarize the effects of BCAAs supplementation on the attenuation of plasma muscle damage markers and soreness after resistance exercise in trained males. The main findings are that BCAAs supplementation could mitigate the CK efflux at all follow-up times after exercise (<24, 24, 48 h) and reduce the muscle soreness (VAS) at <24 h post-exercise, whilst there is not a further effect on LDH reduction at any follow-up time (24 and 48 h). Distinct exercise response patterns in the plasma muscle damage markers LDH and CK may suggest their implication in the early and late stages of inflammation. Muscle damage induces inflammation, which is involved with clearance of damaged cells by phagocytosis (within 24 h), followed by a protracted phase of cell regeneration for several days before resolution of inflammation [6]. The significant treatment effect highlights the role of BCAAs in accelerating muscle regeneration after resistance exercise. Muscle regeneration is a long process and demands nitrogen source, which could only be obtained from protein and amino acids, not carbohydrate and fat. Therefore, it is likely that the significant effect of BCAAs supplementation on attenuating CK elevation is associated with shortening the second, regenerative stage of inflammation by supplying nitrogen source.

To circumvent the potential influence of estrogen fluctuation on the muscle damage outcomes, this meta-analysis surveyed only to the studies involving trained males. Differences in physical fitness status, EIMD, and combination of BCAAs with other essential amino acids or vitamins were also restricted. To the best of our knowledge, no meta-analysis has been previously proposed with this restrictive set of criteria. Most of previous meta-analyses [23,24,25] reviewing the effect of BCAAs on muscle damage did not exclude the factors of sex, physical fitness statuses, types of exercise, and supplementation interventions.

Consistently with previous meta-analyses [23,24,25], a reduction in LDH did not emerged at any follow-up time after exercise. LDH is a marker for damage of contractile elements in the muscle [24] occurring during the early stage of inflammation [6]. The increase in the serum concentration of LDH may depend on exercise conditions, the primary site of muscle damage, and training status [23]. However, due to the limited number of studies only focused on resistance exercise, it is not possible to provide definite conclusions on the efficacy of BCAAs on LDH reduction after EIMD.

The current meta-analysis demonstrated the positive effect of ingestion of BCAAs before and after exercise in reducing the efflux of CK at <24, 24, and 48 h after exercise. Previous studies demonstrated higher levels of CK in healthy individuals (but not highly trained) compared to athletes or trained participants, suggesting an association between physical fitness status and CK release [35,36,42]. The result of the current meta-analysis is in accordance with previous meta-analyses regarding the effect of BCAAs observed at the follow-up 24 h after exercise [25] and both <24 and 24 h [23]. Previous studies illustrated that CK was significantly reduced in the BCAAs group compared to the placebo group [34,35,40,41]. It has been suggested that the effect of BCAAs in the reduction of CK release and magnitude of muscle damage could be explained by the greater bioavailability of nitrogen source and the maintenance of the membrane integrity in the secondary phase of muscle damage after eccentric exercise [34,40].

Contrasting results are available for the follow-up 48 h, since the current meta-analysis found a positive effect of BCAAs, whilst a reduction in CK release did not emerge in previous meta-analyses [23,25]. The inconsistency in results at 48 h after exercise might be explained by the limited number of studies included in the meta-analysis of Rahimi and colleagues [23]. Furthermore, the fitness status and age of the recruited participants can modulate the magnitude of the muscle damage [36,42] and, therefore, the effect of BCAAs supplementation during the time-course of recovery [34]. Moreover, gender differences in serum enzyme activity could further influence the magnitude of the effects [33]. However, the entire response pattern of CK remains unclear. Peak levels could be seen anywhere from 24 to 96 h and could be influenced by individual factors and exercise variables [11,12]. Unless for two experimental conditions (low dosage [36] and control group [41]), in all the remaining conditions the peak of CK has been found at 24 h. However, only 2 studies investigate the CK at follow-up ≥72 h [34,41]. Therefore, more research should investigate the entire response pattern of CK after resistance exercise to clarify its role during the early and/or late stage of inflammation.

The current meta-analysis confirmed the beneficial effect of BCAAs on muscle soreness at <24 h but no further effects at 24 and 48 h after exercise. The result is in disagreement with a previous meta-analysis [25], which showed a significant decrease of muscle soreness at 24, 48, and 72 h. Previous evidence showed that the muscle soreness derives from the inflammation of perimysium or epimysium [43] and from the tissue breakdown products which stimulates the nociceptors in the muscles so that the pain sensation increases [2]. The possible explanation for the effect of BCAAs in mitigating muscle soreness could be the action of glutamine. Glutamine is an abundant free amino acid in the plasma and skeletal muscle that is related to protein synthesis [44]. Generally, glutamine is highly used by the inflammatory and damaged cells to attenuate the magnitude of damage and decrease the soreness. BCAAs can be also transaminated to glutamate in order to enhance glutamine production [18,45]. Nevertheless, the mechanism of the effect of BCAAs on muscle soreness remains to be elucidated.

Considering the available evidence, it is not possible to define a unique strategy for the BCAAs supplementation as the dosage of BCAAs varied across studies. In fact, only two studies investigated the effect of different dosages of BCAAs, showing the superior effect of a high dose of BCAAs (18 g) on muscle soreness reduction compared to a low dose (6 g) [38]. Conversely, a low dose (210 mg/kg) of BCAAs showed a greater effect in reducing LDH compared to a high dose (450 mg/kg) [36]. Though these results are unexplained, the effective dosage of BCAAs for the reduction of muscle damage and soreness remains unclear. Moreover, the ratio of BCAAs (i.e., leucine, isoleucine, and valine), timing (i.e., pre- or post-exercise, or a combination of both), and duration of supplementation (i.e., short or long) need to be considered in order to derive proper conclusions and provide recommendation for practitioners.

This meta-analysis focused only on BCAAs supplementation and resistance exercise, even though other protein sources are commonly used and explored. Whey protein, having a high availability of BCAAs, has been investigated for its efficiency in stimulating muscle protein synthesis [15,18,19]. However, previous meta-analyses demonstrated small to moderate effects of whey protein on muscle function during the temporal recovery from <24 to 96 h after resistance exercise [46], and a reduction of myoglobin and CK levels considering different form of exercises [47]. However, whey protein failed to reduce CK and muscle soreness during recovery until 96 h, either as a pre- or post-exercise supplementation during eccentric isokinetic contractions [48]. Moreover, a previous investigation evaluating healthy individuals for 14 days following a unilateral eccentric contraction-based resistance exercise demonstrated a positive effect of whey protein on muscle strength and LDH, but not on CK [49]. The available evidence is not sufficient to demonstrate a clear effect of whey protein in reducing muscle damage even though it is difficult to compare findings from different study designs, as well as it is not possible to have a comprehensive comparison among different protein sources. In addition, several protein sources are all capable of activating muscle protein synthesis [19]. Nevertheless, the use of BCAAs supplementation can still be recommended among athletes and highly trained individuals, who are exposed to high and frequent training loads to reduce the magnitude of EIMD and to accelerate the time-course of recovery after resistance exercise.

The current meta-analysis has some limitations that need to be addressed. Firstly, the number of included studies did not allow to analyze the effect of BCAAs at follow-up times 72 and 96 h. Secondly, the effects on muscle soreness at 24 and 48 h need to be interpreted at the light of the single study demonstrating no effect and influencing the overall effect [39]. Therefore, the final result on muscle soreness reduction should be considered not conclusive. Thirdly, precise information of the daily protein intake was not available, even though it is considered a critical factor in studies investigating the supplementation of nitrogen source.

This meta-analysis might also serve as a guidance for future research. In light of the current results, more studies are necessary for understanding the effects of BCAAs in resistance-trained males: (a) over a prolonged time-course of recovery (until 72 or 96 h); (b) comparing high and low dosage; (c) considering different timing of supplementation (pre- or post-exercise, or a combination of both); (d) based on preload (i.e., short or long duration); and (e) based on different ratios of leucine, isoleucine, and valine. Furthermore, the integration of a subjective measurement of muscle soreness (i.e., VAS) with objective measurements of plasma muscle damage markers should be always pursued for a complete understanding of EIMD.

However, the potential benefit of BCAAs supplementation could be investigated in patients with specific diseases causing an elevated muscle damage and consequently plasma CK concentration, like muscular dystrophies [50].

## 5. Conclusions

The present meta-analysis demonstrated that the BCAAs supplementation has the potential effect to decrease the CK efflux and attenuate muscle soreness when the analysis is restricted to only trained males after resistance exercise, whilst there is no further benefit on the reduction of LDH. This result implicates that BCAAs supplementation has no effect on preventing muscle damage but accelerates the resolution of inflammation by activating cell regeneration. Therefore, BCAAs could be used as an effective strategy to reduce the magnitude of EIMD and to accelerate the time-course of recovery after resistance exercise.

## Figures and Tables

**Table 1:** PICOS model used to conduct the meta-analysis

Parameter	Inclusion Criteria
Population	Trained males Athletes
Intervention	BCAAs supplementation
Comparators	Placebo or control group
Outcomes	LDH, CK, VAS
Study design	Randomized Controlled Trials

BCAAs = Branched-Chain Amino Acids, CK = Creatine Kinase, LDH = Lactate Dehydrogenase, VAS = Visual Analog Scale.

**Table 2:** Study characteristics.

Author, Year	Participants Exp, Con (Study design)	Exercise Intervention	Supplementation Strategies	Follow-up Times	Outcome Measured
Barzegari, 2011 [33]	20, 20 (Parallel)	4 sets × 10 reps at 80% 1RM of seven multi-joints exercises	68 mg/kg for 6 days before and 450 mg/kg at pre- and post-exercise LEU/ISO/VAL (2:1:1) Placebo: Dextrin	Pre, 24, 48 h	CK LDH
Howatson, 2012 [34]	6, 6 (Parallel)	5 sets × 20 reps of drop jumps	20 g for 11 days and 20 g at pre- and post-exercise LEU/ISO/VAL (2:1:1) Placebo: Artificial sweetener	Pre, 24, 48, 72, 96 h	CK VAS
Atashak, 2012 [35]	20, 20 (Parallel)	7 reps of 100% 1RM until volitional fatigue	200 mg/kg at pre-exercise LEU/ISO/VAL (2:1:1) Placebo: Omega-3 Fatty acids	Pre, Post-Im, 1, 2, 24 h	CK
Amirsasan, 2014 [36]	10 High dose 10 Low dose 9 Con (Parallel)	3 sets × 10 reps at 80% 1RM of seven exercises multi and single-joint	68 mg/kg for 6 days before and 210 mg/kg (low) or 450mg/kg (high) at pre- and post-exercise LEU/ISO/VAL (2:1:1) Placebo: Dextrin	Pre, 24, 48 h	CK LDH
Gee, 2016 [37]	11 (Crossover)	4 sets × 8 reps at 80% 1RM of multi-joint barbell exercises	10 g at pre- and post-exercise LEU/ISO/VAL (2:1:1) Placebo: Apple and blackcurrant juice	Pre, 24 h	VAS
Dorrell, 2016 [38]	5 High/Low dose (Crossover)	4 sets × 8 reps at 75% 1RM of multi-joint barbell exercises	6 g or 18 g at pre- and post-exercise LEU/ISO/VAL (2:1:1) Placebo: Artificial sweetener	Pre, Post-Im	VAS
Kephart, 2016 [39]	15, 15 (Parallel)	10 sets × 5 reps at 80% 1RM of barbell back squat	12 g for 3 consecutive exercise days LEU/ISO/VAL (3:1:2) Placebo: Carbohydrate	Pre, 24, 48 h	VAS
Shenoy, 2017 [40]	10, 10 (Parallel)	5 sets × 20 reps of drop jump	20 g for 4 weeks at pre-exercise LEU/ISO/VAL (2:1:1) Placebo: Aspartame	Pre, 24, 48 h	CK
VanDusseldorp, 2018 [41]	10, 10 (Parallel)	10 sets × 8 reps at 70% 1RM of squat + 5 sets × 20 reps split jump	0.22 g/kg/day for 8 days at pre-exercise LEU/ISO/VAL (3:1:2) Placebo: Maltodextrin	Pre, 4, 24, 48, 72 h	CK VAS

1RM = 1 Repetition Maximum, CK = Creatine Kinase, Con = Control or Placebo group, Exp = Experiment group, LDH = Lactate Dehydrogenase, LEU/ISO/VAL = leucine/isoleucine/valine, reps = repetitions, VAS = Visual Analog Scale.

## Data Availability

No new data were created or analyzed in this study. Data sharing is not applicable to this article.

## References

[1] Clarkson P.M., Hubal M.J. (2002). Exercise-induced muscle damage in humans. Am. J. Phys. Med. Rehabil..

[2] Proske U., Morgan D.L. (2001). Muscle damage from eccentric exercise: Mechanism, mechanical signs, adaptation and clinical applications. J. Physiol..

[3] Nosaka K., Sakamoto K. (2001). Effect of elbow joint angle on the magnitude of muscle damage to the elbow flexors. Med. Sci. Sports Exerc..

[4] Chapman D., Newton M., Sacco P., Nosaka K. (2006). Greater muscle damage induced by fast versus slow velocity eccentric exercise. Int. J. Sports. Med..

[5] Howatson G., van Someren K.A. (2008). The prevention and treatment of exercise-induced muscle damage. Sports Med..

[6] Tidball J.G. (2017). Regulation of muscle growth and regeneration by the immune system. Nat. Rev. Immunol..

[7] Byrne C., Twist C., Eston R. (2004). Neuromuscular function after exercise-induced muscle damage. Sports Med..

[8] Coombes J., McNaughton L. (2000). Effects of branched-chain amino acid supplementation on serum creatine kinase and lactate dehydrogenase after prolonged exercise. J. Sports Med. Phys. Fitness.

[9] Koba T., Hamada K., Sakurai M., Matsumoto K., Hayase H., Imaizumi K., Tsujimoto H., Mitsuzono R. (2007). Branched-chain amino acids supplementation attenuates the accumulation of blood lactate dehydrogenase during distance running. J. Sports Med. Phys. Fitness.

[10] Paulsen G., Ramer Mikkelsen U., Raastad T., Peake J.M. (2012). Leucocytes, cytokines and satellite cells: What role do they play in muscle damage and regeneration following eccentric exercise?. Exerc. Immunol. Rev..

[11] Brancaccio P., Limongelli F., Maffulli N. (2006). Monitoring of serum enzymes in sport. Br. J. Sports Med..

[12] Koch A., Pereira R., Machado M. (2014). The creatine kinase response to resistance exercise. J. Musculoskelet. Neuronal. Interact..

[13] Yu J.-G., Fürst D.O., Thornell L.-E. (2003). The mode of myofibril remodelling in human skeletal muscle affected by DOMS induced by eccentric contractions. Histochem. Cell. Biol..

[14] Blomstrand E., Eliasson J., Karlsson H.K., Köhnke R. (2006). Branched-chain amino acids activate key enzymes in protein synthesis after physical exercise. J. Nutr..

[15] Gorissen S.H., Phillips S.M. (2019). Branched-chain amino acids (leucine, isoleucine, and valine) and skeletal muscle. Nutrition and Skeletal Muscle.

[16] Millward D.J., Layman D.K., Tomé D., Schaafsma G. (2008). Protein quality assessment: Impact of expanding understanding of protein and amino acid needs for optimal health. Am. J. Clin. Nutr..

[17] Harper A., Miller R., Block K. (1984). Branched-chain amino acid metabolism. Annu. Rev. Nutr..

[18] Nicastro H., Da Luz C.R., Chaves D.F.S., Bechara L.R.G., Voltarelli V.A., Rogero M.M., Lancha A.H. (2012). Does branched-chain amino acids supplementation modulate skeletal muscle remodeling through inflammation modulation? Possible mechanisms of action. J. Nutr. Metab..

[19] Jäger R., Kerksick C.M., Campbell B.I., Cribb P.J., Wells S.D., Skwiat T.M., Purpura M., Ziegenfuss T.N., Ferrando A.A., Arent S.M. (2017). International society of sports nutrition position stand: Protein and exercise. J. Int. Soc. Sports. Nutr..

[20] Tom A., Nair K.S. (2006). Assessment of branched-chain amino acid status and potential for biomarkers. J. Nutr..

[21] Da Luz C.R., Nicastro H., Zanchi N.E., Chaves D.F., Lancha A.H. (2011). Potential therapeutic effects of branched-chain amino acids supplementation on resistance exercise-based muscle damage in humans. J. Int. Soc. Sports Nutr..

[22] Fouré A., Bendahan D. (2017). Is branched-chain amino acids supplementation an efficient nutritional strategy to alleviate skeletal muscle damage? A systematic review. Nutrients.

[23] Rahimi M.H., Shab-Bidar S., Mollahosseini M., Djafarian K. (2017). Branched-chain amino acid supplementation and exercise-induced muscle damage in exercise recovery: A meta-analysis of randomized clinical trials. Nutrition.

[24] Hormoznejad R., Zare J.A., Mansoori A. (2019). Effect of BCAA supplementation on central fatigue, energy metabolism substrate and muscle damage to the exercise: A systematic review with meta-analysis. Sport Sci. Health.

[25] Rahimlou M., Ahmadi A.H.R., Palimi E., Mahdipour M., Poodeh B.M. (2020). Reduction of muscle injuries and improved post-exercise recovery by branched-chain amino acid supplementation: A systematic review and meta-analysis. J. Nutr. Fasting Health.

[26] Dieli-Conwright C.M., Spektor T.M., Rice J.C., Schroeder E.T. (2009). Hormone therapy attenuates exercise-induced skeletal muscle damage in postmenopausal women. J. Appl. Physiol..

[27] Page M.J., McKenzie J.E., Bossuyt P.M., Boutron I., Hoffmann T.C., Mulrow C.D., Shamseer L., Tetzlaff J.M., Akl E.A., Brennan S.E. (2021). The PRISMA 2020 statement: An updated guideline for reporting systematic reviews. Brit. Med. J..

[28] Brown P.B.K., Chalkidou K., Chalmers I., Clarke M., Fenton M., Forbes C., Glanville J., Hicks N.J., Moody J. (2006). How to formulate research recommendations. Brit. Med. J..

[29] Kadic A.J., Vucic K., Dosenovic S., Sapunar D., Puljak L. (2016). Extracting data from figures with software was faster, with higher interrater reliability than manual extraction. J. Clin. Epidemiol..

[30] Sterne J.A., Savović J., Page M.J., Elbers R.G., Blencowe N.S., Boutron I., Cates C.J., Cheng H.-Y., Corbett M.S., Eldridge S.M. (2019). RoB 2: A revised tool for assessing risk of bias in randomised trials. Brit. Med. J..

[31] Higgins J.P.T., Chandler J., Cumpston M., Li T., Page M.J., Welch V.A. (2021). Cochrane Handbook for Systematic Reviews of Interventions Version 6.2 (updated February 2021).

[32] Higgins J.P.T., Thompson S.G., Deeks J.J., Altman D.G. (2003). Measuring inconsistency in meta-analyses. Brit. Med. J..

[33] Barzegari A. (2011). Effect of 450 Mg.kg^−1^ branched-chain amino acid supplement on muscle serum damage indices. World Appl. Sci. J..

[34] Howatson G., Hoad M., Goodall S., Tallent J., Bell P.G., French D.N. (2012). Exercise-induced muscle damage is reduced in resistance-trained males by branched chain amino acids: A randomized, double-blind, placebo controlled study. J. Int. Soc. Sports. Nutr..

[35] Atashak S., Baturak K. (2012). The effect of BCAA supplementation on serum C-reactive protein and creatine kinase after acute resistance exercise in soccer players. Ann. Biol. Res..

[36] Amirsasan R., Nikookheslat S., Sari-Sarraf V., Kaveh B., Letafatkar A. (2014). The effects of two different dosages of BCAA supplementation on a serum indicators of muscle damage in wrestlers. Int. J. Wrestl. Sci..

[37] Gee T.I., Deniel S. (2016). Branched-chain aminoacid supplementation attenuates a decrease in power-producing ability following acute strength training. J. Sports Med. Phys. Fitness.

[38] Dorrell H.F., Gee T.I. (2016). The acute effects different quantities of branched-chain amino acids have on recovery of muscle function. Sports Nutr. Ther..

[39] Kephart W.C., Mumford P.W., McCloskey A.E., Holland A.M., Shake J.J., Mobley C.B., Jagodinsky A.E., Weimar W.H., Oliver G.D., Young K.C. (2016). Post-exercise branched chain amino acid supplementation does not affect recovery markers following three consecutive high intensity resistance training bouts compared to carbohydrate supplementation. J. Int. Soc. Sports Nutr..

[40] Shenoy S., Dhawan M., Sandhu J.S. (2017). Effect of chronic supplementation of branched chain amino acids on exercise-induced muscle damage in trained athletes. J. Sports Sci..

[41] VanDusseldorp T.A., Escobar K.A., Johnson K.E., Stratton M.T., Moriarty T., Cole N., McCormick J.J., Kerksick C.M., Vaughan R.A., Dokladny K. (2018). Effect of branched-chain amino acid supplementation on recovery following acute eccentric exercise. Nutrients.

[42] Fernandes J.F.T., Lamb K.L., Twist C. (2019). Exercise-induced muscle damage and recovery in young and middle-aged males with different resistance training experience. Sports.

[43] Malm C. (2001). Exercise-induced muscle damage and inflammation: Fact or fiction?. Acta Physiol. Scand..

[44] Raizel R., Tirapegui J. (2018). Role of glutamine, as free or dipeptide form, on muscle recovery from resistance training: A review study. Nutrire.

[45] Street B., Byrne C., Eston R. (2011). Glutamine supplementation in recovery from eccentric exercise attenuates strength loss and muscle soreness. J. Exerc. Sci. Fit..

[46] Davies R.W., Carson B.P., Jakeman P.M. (2018). The effect of whey protein supplementation on the temporal recovery of muscle function following resistance training: A systematic review and meta-analysis. Nutrients.

[47] Lam F.C., Khan T.M., Faidah H., Haseeb A., Khan A.H. (2019). Effectiveness of whey protein supplements on the serum levels of amino acid, creatinine kinase and myoglobin of athletes: A systematic review and meta-analysis. Syst. Rev..

[48] White J.P., Wilson J.M., Austin K.G., Greer B.K., St John N., Panton L.B. (2008). Effect of carbohydrate-protein supplement timing on acute exercise-induced muscle damage. J. Int. Soc. Sports. Nutr..

[49] Cooke M.B., Rybalka E., Stathis C.G., Cribb P.J., Hayes A. (2010). Whey protein isolate attenuates strength decline after eccentrically-induced muscle damage in healthy individuals. J. Int. Soc. Sports. Nutr..

[50] Lott D.J., Taivassalo T., Cooke K.D., Park H., Moslemi Z., Batra A., Forbes S.C., Byrne B.J., Walter G.A., Vandenborne K. (2021). Safety, feasibility, and efficacy of strengthening exercise in Duchenne muscular dystrophy. Muscle Nerve.

